# Validation of Predictive Models for Autoimmune Encephalitis-Related Antibodies to Cell-Surface Proteins Expressed in Neurons: A Retrospective Study Based in a Hospital

**DOI:** 10.3389/fneur.2021.601761

**Published:** 2021-05-14

**Authors:** Siqi Ding, Jiaoni Gong, Jiahe Lin, Yi Wang, Yingjie Hua, Xueying Li, Yanru Du, Niange Xia, Zhenguo Zhu, Xinshi Wang, Rongyuan Zheng, Huiqin Xu

**Affiliations:** ^1^Department of Neurology, The First Affiliated Hospital of Wenzhou Medical, University, Wenzhou, China; ^2^Department of Preventive Medicine, School of Public Health and Management, Wenzhou Medical University, Wenzhou, China

**Keywords:** autoimmune encephalitis, antibody, antibody prevalence in epilepsy and encephalopathy (APE^2^ score), predictive model, validation

## Abstract

**Objective:** Autoimmune encephalitis (AE) is a severe but treatable autoimmune disorder that is diagnosed by antibody (Ab) testing. However, it is unrealistic to obtain an early diagnosis in some areas since the Ab status cannot be immediately determined due to time and technology restrictions. In our study, we aimed to validate the Antibody Prevalence in Epilepsy and Encephalopathy (APE^2^) score among patients diagnosed with possible AE as a predictive model to screen AE patients with antibodies to cell-surface proteins expressed in neurons.

**Methods:** A total of 180 inpatients were recruited, and antibodies were detected through serological and/or cerebrospinal fluid (CSF) evaluations. The APE^2^ score was used to validate the predictive models of AE with autoantibodies.

**Results:** The mean APE^2^ score in the Ab-positive cases was 7.25, whereas the mean APE^2^ score in the Ab-negative cases was 3.18 (*P* < 0.001). The APE^2^ score had a receiver operating characteristic (ROC) area under the curve of 0.924 [*P* < 0.0001, 95% confidence interval (CI) = 0.875–0.973]. With a cutoff score of 5, the APE^2^ score had the best psychometric properties, with a sensitivity of 0.875 and a specificity of 0.791.

**Conclusion:** The APE^2^ score is a predictive model for AE with autoantibodies to cell-surface proteins expressed in neurons and was validated and shown to have high sensitivity and specificity in our study. We suggest that such a model should be used in patients with a suspected diagnosis of AE, which could increase the detection rate of Abs, reduce testing costs, and help patients to benefit from treatment quickly.

## Introduction

Autoimmune encephalitis (AE) is an immune-mediated neurological disorder characterized by rapidly progressive central nervous system (CNS) symptoms that is associated with specific autoantibodies targeting cell-surface neuronal antigens (1). AE is classified into different subtypes according to the neuronal antigens targeted by the autoantibodies found in the serum and/or cerebrospinal fluid (CSF) of patients, each of which has different clinical characteristics and outcomes (1, 2). The identified forms of AE might be associated with antibodies (Abs) against intracellular antigens, synaptic receptors, ion channels, or cell-surface proteins, according to the location of these specific autoantibodies (3).

As more related cases have been identified in the past 10 years, there has been increasing interest in the pathogenesis and clinical features of AE, especially in patients with Abs to cell-surface proteins expressed in neurons, including antibodies against synaptic receptors and antibodies targeting ion channels and cell-surface proteins, who have an effective response to immunosuppressive therapies and who respond well to immunosuppressive therapies (4). Abs to N-methyl-d-aspartate receptor (NMDAR), gamma-aminobutyric acid A receptor (GABAAR), gamma-aminobutyric acid B receptor (GABABR), alpha-amino-3-hydroxy-5-methyl-4-isoxazolepropionic acid receptor (AMPAR), metabotropic glutamate receptor 5 (mGluR5), dopamine 2 receptor, leucine-rich glioma-inactivated 1 (LGI1), contactin-associated protein-like 2 (Caspr2), and dipeptidyl-peptidase-like protein 6 (DPPX) were identified in patients and were associated with various clinical manifestations (5).

The current diagnostic criteria for AE depend heavily on Ab testing and responses to immunotherapy (6). However, the identification of the different forms of Abs involved with AE is a complicated process. The diagnosis of AE should be suspected based on Ab identification, as well as combination of disease signs and symptoms (3). In addition, some patients with AE may not respond to immunotherapy or may require intensive and long-term treatment, which is unavailable in most health care systems unless a diagnosis has been previously established (2). However, rapid access to gold standard diagnostic cell-based assays is not universally available, especially in some areas where medical conditions are limited. An Ab prediction model that is not based on Ab detection not only is helpful for early diagnosis but also can save medical resources and reduce the economic burden of patients. These issues prompted researchers to explore methods for the early diagnosis of AE and to establish predictive models for the detection of autoantibodies based on the clinical presentation and initial neurologic evaluations prior to Ab testing.

Recently, an Antibody Prevalence in Epilepsy and Encephalopathy (APE^2^) score was described as a model for predicting the detection of neural-specific autoantibodies based on clinical characteristics; this model was validated and had high sensitivity and specificity for identifying patients with cognitive decline (7–9). AE usually presents with the subacute onset of memory deficits or an altered mental status, which may or may not be accompanied by other symptoms (2). Therefore, we aimed to validate the APE^2^ scoring system as a predictive model to screen AE patients with antibodies to cell-surface proteins expressed in neurons among patients diagnosed with possible AE.

## Methods

### Subjects

We conducted a retrospective review of inpatients diagnosed with possible AE between June 2014 and June 2019 at the First Affiliated Hospital of Wenzhou Medical University, China. The levels of AE autoantibodies of every inpatient were detected through serological and/or CSF evaluations. Patients were included in the study based on the following criteria: (1) onset of working memory deficits (short-term memory loss), altered mental status, or psychiatric symptoms; (2) at least one of the following: new focal CNS findings, seizures not explained by a previously known seizure disorder, CSF pleocytosis [white blood cell (WBC) count of more than 5 cells per mm], and magnetic resonance imaging (MRI) features suggestive of encephalitis; and (3) reasonable exclusion of alternative causes. Patients with incomplete medical record information were excluded.

### Ab Evaluations

All serum and CSF specimens were screened by standardized indirect immunofluorescence assays (IFAs) and cell-based assays (CBAs) using human embryonic kidney (HEK) 293 cells transfected with appropriate expression plasmids to confirm IgGs specific for NMDAR, AMPA1, AMPA2, LGI1, CASPR2, GABABR, and DPPX. The initial dilution titers of CSF and serum were 1:1 and 1:10, respectively.

### Data Collection

Data were gathered from each patient, which included basic demographic information (age and sex), the duration of symptoms before hospitalization, the presence and type of seizures, status epilepticus, mental disorders, sleep disorders, headache, laboratory data, and CSF results (protein, WBCs, glucose, and chloride), MRI, anticardiolipin antibody (ACA), antinuclear antibody (ANA), treatment, intensive care unit (ICU) admission, the need for mechanical ventilation, and the prognosis during hospitalization. The APE^2^ score of each inpatient was evaluated by a neurologist based on the clinical manifestations, and the neurologists were blinded to the actual results of Ab evaluations.

### APE^2^ Score

The initial version of the Antibody Prevalence in Epilepsy (APE) score was established by Dubey as a model to predict the detection of neural Abs in autoimmune epilepsy patients based on clinical manifestations and initial neurologic evaluations (7). An APE score of ≥4 had a sensitivity and specificity of 82.6 and 82.0%, respectively, and can be used as a tool for identifying patients for Ab testing (7). After three modifications, the APE^2^ scoring system has a higher specificity for the prediction of neural autoantibody positivity (from 78 to 84%) among patients with epilepsy, without a loss in sensitivity (98%) (9). The determination of the APE^2^ score in patients with encephalopathy or cognitive decline to predict AE-related Ab positivity showed that an APE^2^ score ≥4 was 99% sensitive and 93% specific for neural-specific Abs (8).

### Statistical Analysis

The data were analyzed using the Statistical Package for the Social Sciences (SPSS version 20.0; SPSS Inc., Chicago, IL, USA). Sociodemographic characteristics and clinical variables are presented as counts, frequency (%), means, and standard deviations. Categorical variables were compared using chi-square tests or Fisher's exact tests. Interval variables were performed using Mann–Whitney *U*-tests. The threshold for statistical significance was set at *P* < 0.05 (two-tailed). The odds ratio (OR) and 95% confidence intervals (CIs) were used to quantify the strength of the associations. Receiver operating characteristic (ROC) curve analysis was performed to assess the sensitivity, specificity, Youden index, and positive and negative predictive values of the APE^2^ score at different cutoff scores.

## Results

Serum, CSF, or both were collected from 238 patients at the onset of the study for the detection of AE-related Ab testing. Thirty-five patients were excluded due to an indefinite diagnosis of encephalitis, whereas 23 patients were excluded due to incomplete records and insufficient clinical data. The final study group consisted of 180 inpatients, among whom 102 patients underwent simultaneous serum and CSF examinations, 38 patients underwent only serum examinations, and 40 patients underwent only CSF examinations. AE-related antibodies in the serum/CSF were detected in 32 of the 180 inpatients, including NMDAR (*n* = 23), LGI1 (*n* = 3), GABABR (*n* = 3), CASPR2 (*n* = 2), and AMPA1 (*n* = 1), which were in accordance with the diagnostic standards of AE. A large number of patients with AE have no well-defined syndrome. According to three types of specific syndromes associated with AE (5), there were anti-NMDAR encephalitis (*n* = 23), limbic encephalitis (*n* = 7), and Morvan's syndrome (*n* = 2). Among these 32 patients, 28 (87.5%) had new-onset seizures, and 28 (87.5%) had mental status changes. Regarding prognosis, 23 (71.9%) patients recovered well, 7 (21.9%) patients did not respond to treatment, and 2 (6.3%) patients were discharged after the deterioration of their condition. Table 1 shows the detailed information of the Ab-positive group and the Ab-negative group, such as the basic demographic information, clinical features, laboratory data, treatment, and prognosis.

**Table 1:** Clinical characteristics of patients in the study.

**Variables**	**Antibody-positive cases (*n* = 32)**	**Antibody-negative cases (*n* = 148)**	***P*-value**
Median age, years (range)	41.09 (14–78)	48.93 (15–87)	0.035
Female *N* (%)	12 (37.5)	56 (37.8)	0.971
Median APE^2^ score	7.25	3.18	0.000
New-onset seizures (%)	28 (87.5)	79 (53.4)	0.000
Duration of symptoms before hospitalization (weeks)			0.293
1–6	31 (96.9)	125 (84.5)	
6–12	1 (3.1)	7 (4.7)	
12–24	0	3 (2.0)	
>24	0	13 (8.8)	
Type of seizure (%)			0.000
No	4 (12.5)	69 (46.6)	
FES	4 (12.5)	20 (13.5)	
GES	12 (37.5)	41 (27.7)	
2nd GES	12 (37.5)	18 (12.1)	
Status epilepticus (%)	11 (34.4)	10 (6.8)	0.000
Mental status changes (%)	28 (87.5)	108 (73)	0.083
Sleep disorder diagnosis (%)	4 (12.5)	18 (12.2)	1.000
Headache (%)	8 (25)	49 (33.1)	0.371
CSF protein >50 mg/dl (%)	6 (18.75)	87 (58.8)	0.000
CSF cell count >5 cells/dl (%)	31 (96.875)	128 (86.5)	0.175
CSF glucose	3.741 (0.7374)	3.941 (1.3785)	0.776
CSF chloride	121.75 (5.798)	122.57 (6.842)	0.716
MRI (T2/FLAIR hyperintensity)			1.000
Normal	17 (53.1)	86 (58.1)	
One or both medial temporal lobes	4 (12.5)	8 (5.4)	
Multifocal in gray matter, white matter	9 (28.1)	28 (18.9)	
Compatible with demyelination or inflammation	2 (6.3)	26 (17.6)	
ACA or ANA			0.344
Positive result	15 (46.9)	90 (60.8)	
Negative result	15 (46.9)	52 (35.1)	
Unmeasured	2 (6.3)	6 (4.1)	
Treatment			0.000
No immunotherapy	3 (9.4)	108 (73)	
Methylprednisolone	11 (34.4)	23 (15.5)	
Immune globulin	3 (9.4)	3 (2.0)	
Combinations of methylprednisolone and immune globulin	14 (43.8)	14 (9.5)	
Other immunotherapy	1 (3.1)	0	
ICU admission	7 (21.9)	19 (12.8)	0.298
Coma	5 (15.6)	19 (12.8)	0.894
Mechanical ventilation	2 (6.3)	14 (9.5)	0.813
Outcome			0.004
Recovered	23 (71.9)	135 (91.2)	
Not responding to the treatment	7 (21.9)	6 (4.1)	
Deterioration	2 (6.3)	7 (4.7)	

*FES, focal epileptic seizure; GES, generalized epileptic seizure; ICU, intensive care unit; CSF, cerebrospinal fluid; ACA, anticardiolipin antibody; ANA, antinuclear antibody*.

In Table 2, we compared the differences in the APE^2^ score between the two groups and evaluated the associations with clinical characteristics. According to the results of univariate analysis, the following items were associated with the Ab-positive group: neuropsychiatric changes (*P* < 0.001, OR = 5.930), autonomic dysfunction (*P* < 0.001, OR = 11.308), viral prodrome (*P* < 0.001, OR = 3.655), faciobrachial dystonic seizures (*P* < 0.001, OR = 6.692), facial dyskinesias (*P* = 0.016, OR = 2.964), refractory epilepsy (*P* < 0.001, OR = 7.367), and encephalitis and a cancer diagnosis within 5 years of encephalopathy or cognitive dysfunction (*P* = 0.018, OR = 4.552). Conversely, there were no associations between Ab positivity and subacute encephalopathy/new-onset epilepsy (*P* = 0.113), CSF inflammatory changes (P = 0.564), or MRI changes (*P* = 0.702). The mean APE^2^ scores (Table 1) were 7.25 in the Ab-positive group and 3.18 in the Ab-negative group (*P* < 0.001).

**Table 2:** Components of the APE^2^ score.

**Components**	**Value (total: 18)**	**Antibody-positive cases (*n* = 32)**	**Antibody-negative cases (*n* = 148)**	**OR (95% CI)**	***P*-value**
1. New-onset, rapidly progressive mental status changes that developed over 1–6 weeks or new-onset seizure activity (within 1 year of evaluation)	(+1)	31 (96.9)	125 (84.5)	4.769 (0.682–33.334)	0.113
2. Neuropsychiatric changes; agitation, aggressiveness, emotional lability	(+1)	26 (81.3)	50 (33.8)	5.930 (2.568–13.694)	0.000
3. Autonomic dysfunction [sustained atrial tachycardia or bradycardia, orthostatic hypotension (≥20 mmHg fall in systolic pressure or ≥10 mmHg fall in diastolic pressure within 3 min of quiet standing), hyperhidrosis, persistently labile blood pressure, ventricular tachycardia, cardiac asystole, or gastrointestinal dysmotility]	(+1)	21 (65.6)	5 (3.4)	11.308 (6.209–20.595)	0.000
4. Viral prodrome (rhinorrhea, sore throat, low grade fever) to be scored in the absence of underlying systemic malignancy within 5 years of neurological symptom onset	(+2)	15 (46.9)	20 (13.5)	3.655 (2.030–6.582)	0.000
5. Faciobrachial dystonic seizures	(+3)	6 (18.75)	0	6.692 (4.695–9.540)	0.000
6. Facial dyskinesias to be scored in the absence of faciobrachial dystonic seizures	(+2)	6 (18.75)	7 (4.7)	2.964 (1.494–5.882)	0.016
7. Seizure refractory to at least two anti-seizure medications	(+2)	17 (53.1)	7 (4.7)	7.367 (4.270–12.709)	0.000
8. CSF findings consistent with inflammation (elevated CSF protein >50 mg/dl and/or lymphocytic pleocytosis >5 cells/μl, if the total number of CSF RBC is <1,000 cells/μl)	(+2)	21 (65.6)	89 (60.1)	1.215 (0.625–2.363)	0.564
9. Brain MRI suggesting encephalitis (T2/FLAIR hyperintensity restricted to one or both medial temporal lobes, or multifocal in gray matter, white matter, or both compatible with demyelination or inflammation)	(+2)	6 (18.8)	21 (14.2)	1.308 (0.595–2.875)	0.702
10. Systemic cancer diagnosed within 5 years of neurological symptom onset (excluding cutaneous squamous cell carcinoma, basal cell carcinoma, brain tumor, cancer with brain metastasis)	(+2)	3 (9.4)	1 (0.7)	4.552 (2.361–8.774)	0.018

**Figure 1:**
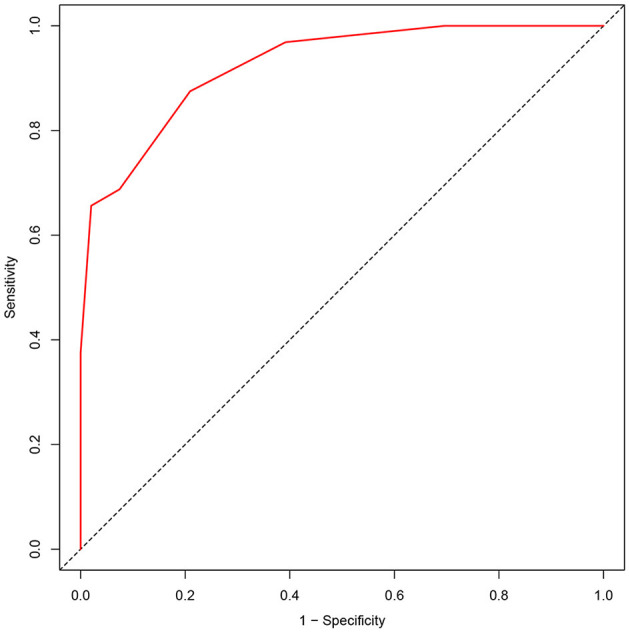
ROC analyses of the APE^2^ score. ROC, receiver operating characteristic.

The area under the ROC of the APE^2^ score was 0.924 (*P* < 0.0001, 95% CI = 0.875–0.973) (Figure 1). As a model to predict Ab-positive AE, an APE^2^ score ≥5 had a sensitivity of 0.875, a specificity of 0.791, and a Youden index of 0.666 (Table 3). The Hosmer–Lemeshow test *P*-value was 0.827 in our study.

**Table 3:** ROC and diagnostic efficiency of the APE^2^ score for the diagnosis of AE with autoantibodies to cell-surface proteins expressed in neurons.

**Cutoff score**	**Sensitivity**	**Specificity**	**PPV**	**NPV**	**YI**
4	0.969	0.608	0.348	0.989	0.577
5	0.875	0.791	0.475	0.967	0.666
6	0.688	0.926	0.667	0.932	0.614
7	0.656	0.98	0.875	0.929	0.636

*PPV, positive predictive value; NPV, negative predictive value; YI, Youden index*.

**Figure 2:**
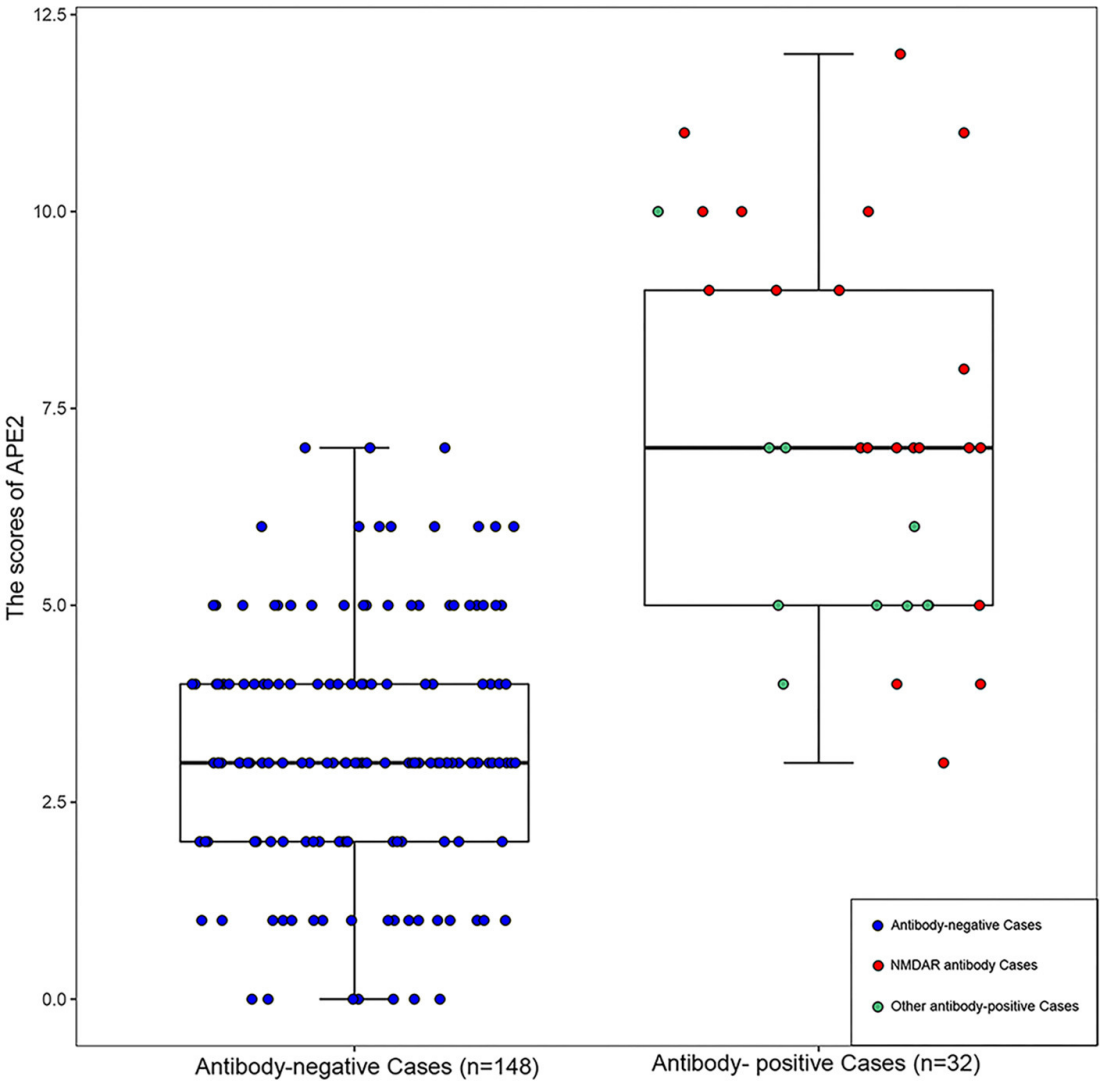
The scores distribution of APE^2^ in each patient. NMDAR antibody cases: *n* = 23; other antibody-positive cases including LGI1 (*n* = 3), GABABR (*n* = 3), CASPR2 (*n* = 2), and AMPA1 (*n* = 1).

We also examined the score distribution of each patient in Figure 2 to perform further comparisons. NMDAR Ab cases were compared separately from other Ab cases (such as LGI1, GABABR, CASPR2, and AMPA1) in the 32 patients with positive Abs, and the mean APE^2^ scores were 7.74 and 6, respectively (*P* = 0.058).

## Discussion

This retrospective study was based in a hospital. Among the patients diagnosed with possible AE, 17.8% had AE-related Abs, which were not present at a low rate. This study suggests that AE among hospitalized patients with encephalopathy is not well-recognized. As a prediction model for AE, the APE^2^ scoring system has high sensitivity and specificity, which makes this prediction model more widely used.

The clinical manifestations of AE varied. A large number of patients with AE do not present with a defined syndrome (10). Autoantibody testing is the most clear and important way to determine the diagnosis of these different conditions, and we can define comorbidities, tumor associations, and prognosis according to these testing results (2). However, immune-mediated limbic encephalitis can occur without detectable autoantibodies, which means that the absence of autoantibodies does not exclude the possibility of AE. In our study, a patient with abnormal behavior and apathy as initial symptoms responded well to immunotherapy. However, the previous two autoimmunity antibodies tested in the CSF were negative, and NMDAR antibodies were found in the third test. Hence, more methods are needed to screen AE patients with antibodies to cell-surface proteins expressed in neurons in clinical practice. As a predictive model with high sensitivity and specificity, the APE^2^ score is well-defined, easily measured, routinely available, and can be used at the bedside. Therefore, the use of the APE^2^ score during diagnosis to screen out suspicious patients is beneficial.

The inclusion criteria used in our study were based on Graus's criteria. However, the duration of symptoms before hospitalization was not strictly defined. A subacute disease duration (rapid progression of <3 months) is important to the diagnosis of possible AE. When we reviewed the patients' histories, we found that some patients first presented with a single neurological or psychiatric symptom and developed other symptoms weeks or even months after the onset, which may have led to delays in seeking medical attention. Patients whose disease duration was longer than 3 months but met the other inclusion criteria described above were still included in the study to avoid missing some possible patients with clinical presentations similar to AE.

As shown in our study, among 148 patients without autoantibodies in the serum/CSF, 31 (20.9%) patients had APE^2^ scores ≥5. We cannot exclude the possibility that those patients diagnosed with AE had antibodies against intracellular antigens or synaptic receptors. AE can be caused by the production of several different autoantibodies targeting various neuronal antigens (2). Our study focused on AE with antibodies to cell-surface proteins expressed in neurons for several reasons. First, antibodies against intracellular antigens are defined as paraneoplastic antibodies because they are frequently observed in patients with cancer (11). The most important antibodies included in this group are against Ma2, Hu, and glutamic acid decarboxylase (GAD). AE patients with these antibodies respond significantly worse to immunosuppressive therapies than other AE patients (12). We aimed to predict the presence of antibodies to cell-surface proteins expressed in neurons based on the APE^2^ score and to alleviate the clinical symptoms of patients through timely immunotherapy without the overutilization of these resources, which is very important in the hospital testing. On the other hand, in some areas with limited medical conditions, it is difficult to obtain early diagnosis by determining the status of antibodies. An Ab prediction model that is not based on Ab detection is more practical and convenient for such an applicable range. Moreover, known antibodies might only be the tip of the iceberg, and with advances in detection technology, potential new antibodies targeting the structure of neurons have been identified at an increasing rate.

It should be noted that some AE-related antibodies might be found only in the CSF (13, 14). An observational study showed that antibodies were found in the CSF among 250 patients with anti-NMDAR encephalitis, whereas researchers could find hardly any antibodies in the serum of 14% (36) of these patients (15). Furthermore, when the Abs found were different in the CSF and serum in one patient, although it did not occur in our study, the types of Abs in the CSF were usually determined (16). These findings supported the higher sensitivity of Ab testing in the CSF than in the serum. In our study, 102 patients out of 180 underwent both serum and CSF evaluations. Although 23% of Ab-negative patients did not undergo autoantibody testing in the CSF, which could have led to false-negative results, both CSF and serum were used for neuronal Ab testing in patients in the Ab-positive group. More importantly, among 32 cases, only 2 patients had low Ab titers [NMDAR Ab, 1:10 (all CSF)]; the remaining 30 patients had high titers [range 1:16–1:1,000 (serum or CSF)]. Although the univariate analysis results found no associations between patients with only serum positivity and those with serum and/or CSF positivity (*P* = 0.946), we still recommend that both CSF and serum be used for autoantibody testing in patients with suspected AE.

Statistical analysis showed no significant differences in some items of the APE^2^ score. Firstly, the manifestations of the inpatients included in our study were almost mental disorders or seizures. With the exception of 24 patients with symptoms of encephalopathy for more than 1–6 weeks, the remaining 156 (86.7%) patients scored 1 point on the first item. There were also no statistically significant differences in the items related to CSF inflammatory changes (*P* = 0.564) and MRI changes (*P* = 0.702). A potential reason is that Ab-negative cases included seronegative AE patients. Furthermore, 25.7% of patients only checked the AE-related antibodies to cell-surface proteins expressed in neurons in the serum for this group. Therefore, patients who met the diagnostic criteria for possible AE should be investigated separately. It is possible that the APE^2^ scores might help us identify seronegative AE patients. The weighted points of these three items in the APE^2^ score system are 1, 2, and 2, respectively, which affects the overall scores as well as the sensitivity and specificity of the APE^2^ score.

Our research was based on a relatively small sample. Nevertheless, the small sample did not significantly affect the precision of the study estimates because the incidence rate of AE is rather low. As one of the most common forms of AE (17), 32 patients with anti-NMDAR encephalitis were identified among 761 cases of encephalitis with uncertain etiology in the California Encephalitis Project between September 2007 and February 2011 (18), whereas 32 AE patients were identified among the 180 samples in our study. However, despite the usefulness of our findings, we still hope that a larger sample will be included in future studies. Furthermore, the scores and data for the model analysis for the APE^2^ score were derived from the analysis of a single-center retrospective study. Therefore, the data need to be interpreted with caution, and a perspective prediction with the APE^2^ score is needed in future studies.

## Conclusions

The APE^2^ score was validated as a predictive model with high sensitivity and specificity for AE antibodies to cell-surface proteins expressed in neurons. Based on the evidence presented here, we suggest that patients with an APE^2^ score ≥5 undergo Ab testing. The use of an objective scoring system for suspected AE patients as early as possible can reduce the detection cost, improve the Ab detection rate, and allow timely and effective treatment.

## Data Availability Statement

The raw data supporting the conclusions of this article will be made available by the authors, without undue reservation.

## Ethics Statement

The studies involving human participants were reviewed and approved by the Ethics Committee of the First Affiliated Hospital of Wenzhou Medical University. Written informed consent to participate in this study was provided by the participants' legal guardian/next of kin. Written informed consent was obtained from the individual(s), and minor(s)' legal guardian/next of kin, for the publication of any potentially identifiable images or data included in this article.

## Author Contributions

SD conceived the study and drafted the article. JG collected the clinical data and interpreted the data. JL and YW performed the statistical analyses, transformed the raw data into figures and tables, and compiled the references. HX contributed to the writing of successive versions of the manuscript. All authors contributed with clinical assessment, data acquisition, analysis, diagnosis, and critical revision of the article.

## Conflict of Interest

The authors declare that the research was conducted in the absence of any commercial or financial relationships that could be construed as a potential conflict of interest.
